# Analysis of swimming performance in FINA World Cup long-distance open water races

**DOI:** 10.1186/2046-7648-3-2

**Published:** 2014-01-02

**Authors:** Matthias Alexander Zingg, Christoph Alexander Rüst, Thomas Rosemann, Romuald Lepers, Beat Knechtle

**Affiliations:** 1 Institute of General Practice and for Health Services Research, University of Zurich, Rämistrasse 71, Zurich, 8006 Switzerland; 2 INSERM U1093, Faculty of Sport Sciences, University of Burgundy, Esplanade Erasme, Dijon, 21078 France; 3 Facharzt FMH für Allgemeinmedizin, Gesundheitszentrum St. Gallen, Vadianstrasse 26, St. Gallen, 9001 Switzerland

**Keywords:** Swim, Peak performance, Speed, Age of peak performance

## Abstract

**Background:**

Age and peak performance in ultra-endurance athletes have been mainly investigated in long-distance runners and triathletes, but not for long-distance swimmers. The present study investigated the age and swimming performance of elite ultra-distance swimmers competing in the 5-, 10- and 25-km Fédération Internationale de Natation (FINA) World Cup swimming events.

**Methods:**

The associations of age and swimming speed in elite male and female swimmers competing in World Cup events of 5-, 10- and 25-km events from 2000 to 2012 were analysed using single and multi-level regression analyses.

**Results:**

During the studied period, the swimming speed of the annual top ten women decreased significantly from 4.94 ± 0.20 to 4.77 ± 0.09 km/h in 5 km and from 4.60 ± 0.04 to 4.44 ± 0.08 km/h in 25 km, while it significantly increased from 4.57 ± 0.01 to 5.75 ± 0.01 km/h in 10 km. For the annual top ten men, peak swimming speed decreased significantly from 5.42 ± 0.04 to 5.39 ± 0.02 km/h in 5 km, while it remained unchanged at 5.03 ± 0.32 km/h in 10 km and at 4.94 ± 0.35 km/h in 25 km. The age of peak swimming speed for the annual top ten women remained stable at 22.5 ± 1.2 years in 5 km, at 23.4 ± 0.9 years in 10 km and at 23.8 ± 0.9 years in 25 km. For the annual top ten men, the age of peak swimming speed increased from 23.7 ± 2.8 to 28.0 ± 5.1 years in 10 km but remained stable at 24.8 ± 1.0 years in 5 km and at 27.2 ± 1.1 years in 25 km.

**Conclusion:**

Female long-distance swimmers competing in FINA World Cup races between 2000 and 2012 improved in 10 km but impaired in 5 and 25 km, whereas men only impaired in 5 km. The age of peak performance was younger in women (approximately 23 years) compared to men (about 25–27 years).

## Background

The popularity of professional sports is mainly based on the need of stars or super-humans the average person can admire. In individual sports, record marks as finishing times are of main interest. In swimming, several new world records were set in recent years. Open water long-distance swimming is a rather young sports discipline since the first World Championship over 10 km was held in 1991. Since 1986, a World Cup is held annually in several different countries. The Fédération Internationale de Natation (FINA) [1] does not hold official world records for open water long-distance swimming since weather [2] and water conditions [3] can considerably affect race time in open water long-distance swimming. Open water long-distance swimming can be held with or without wetsuits [4] depending on the length, the water temperature and the individual event. The first long-distance open water swimming event to be introduced in the Olympic Games was the 10 km in Beijing 2008 [5]. However, since water temperatures and weather cannot be influenced in long-distance open water swimming, even finisher times in Olympic Games can hardly be compared.

The age in life when peak performance in sport is accomplished was an often-discussed topic [6-10] as it may predict the point in life when athletic performance starts to decline. In swimming, the age of peak performance has been investigated for freestyle pool swimmers across distances from 50 to 1,500 m [6-10] and for long-distance swims of 26.4 km [11] and 34 km [12] held in open water. In these studies, different findings have been reported for the different distances. Berthelot et al. [6] reported the age of peak swimming performance at approximately 21 years for freestyle swimmers. However, there seemed to be differences in the age of peak swimming speed regarding the length of a swimming performance [9] and the performance level of the athletes [11-13]. Schulz and Curnow [9] reported for professional swimmers that the fastest times in 1,500-m freestyle were achieved at a younger age of about 18 years compared to 50-m freestyle at about 23 years of Berthelot et al. [6] who also investigated professionals in Olympic sport events. Fairbrother [8] investigated US master swimmers and reported that the age of peak swimming speed in 50-m freestyle was in the late 20s and early 30s for men, and at a considerably higher age than reported by Berthelot et al. [6].

The age of peak swimming performance seemed to be dependent upon the race distance. The age of peak swimming speed seemed to decrease with increasing race distance from 50 to 1,500 m and to increase thereafter for longer events and/or distances such as 26.4 km, 12 h and 35 km [10-13]. Recent studies of Eichenberger et al. [11-13] showed that the age of peak performance for long-distance swimmers competing from 26.4 to 35 km was at approximately 30–39 years for both sexes. However, all investigated events were of non-professional nature, where recreational athletes were competing. This raises the question whether the reported increase of the age of peak swimming speed could be due a selection bias of non-professionalism of athletes in these events since studies investigating pool swimmers mainly used elite athletes. Furthermore, ‘The English Channel Swim’ [12] is even not a race as swimmers have to cross the English Channel solo with the help of a personal support crew.

Recent findings suggested that the age of peak swimming speed in elite freestyle swimmers seemed to increase with increasing race distance of more than 1,500-m length [6,9]. Furthermore, the age of the fastest swimmers seemed to increase across years and with increasing length of the race [6,9]. Most authors [6-10] investigated professional athletes in distances up to 1,500 m, but Eichenberger et al. [11-13] investigated elite recreational athletes competing in open water ultra-distance events. In this context, the aim of the present study was to investigate (1) the changes in swimming speed across years in 5-, 10- and 25-km open water events and (2) the change in the age of peak swimming speed of elite long-distance swimmers competing in these events between 2000 and 2012. Based upon existing findings for recreational swimmers, we hypothesized for elite swimmers, firstly, that swimming speed would increase during the studied period and, secondly, that the age of peak swimming speed would be older than that in shorter distances. The knowledge of the age of peak performance in ultra-distance swimming would help coaches and athletes plan an international swimming career of an athlete.

## Methods

All procedures used in the study met the ethical standards of the Swiss Academy of Medical Sciences [14] and were approved by the Institutional Review Board of Kanton St. Gallen, Switzerland with a waiver of the requirement for informed consent of the participants given the fact that the study involved the analysis of publicly available data.

### Data sampling and data analysis

All athletes who ever finished a 5-, 10- and 25-km FINA [1] World Cup open water swimming race between 2000 and 2012 were analysed regarding the association between age and race performance. The data set for this study was obtained from the website of the FINA [1]. To determine the changes in peak swimming speed and in the age of peak swimming speed over time, race times of the annual top and the annual top ten men and women were analysed. To increase the comparability between different distances, all race times were converted to swimming speed in meters per second (m/s) using the equation, swimming speed = race distance / race time. When less than the needed amount of athletes was available in a certain year for a certain distance, that year was excluded from the analysis.

### Statistical analysis

In order to increase the reliability of the data analyses, each set of data was tested for normal distribution and for homogeneity of variances prior to statistical analyses. Normal distribution was tested using a D'Agostino and Pearson omnibus normality test, and homogeneity of variances was tested using a Levene's test. Trends in participation were analysed using regression analysis with ‘straight line’ and ‘exponential growth equation’ models, whereas for each set of data (e.g. each sex), both models where compared using Akaike's Information Criteria (AIC) to decide which model showed the highest probability of correctness. Single and multi-level regression analyses were used to investigate the changes across years in swimming speed and age of the athletes. A hierarchical regression model was used to avoid the impact of a cluster effect on results in case one athlete finished more than once in the annual top or the annual top ten for the analysis of the annual top and the annual top ten finishers regarding the analysis of overall performance and age of peak performance. Furthermore, regression analyses of swimming speed were corrected for age of athletes to prevent misinterpretation of ‘age effect’ as ‘time effect’. Statistical analyses were performed using IBM SPSS Statistics (version 21, IBM SPSS, Chicago, IL, USA) and GraphPad Prism (version 6.01, GraphPad Software, La Jolla, CA, USA). Significance was accepted at *P* < 0.05 (two-sided for *t* tests). Data in the text are given as mean ± standard deviation (SD).

## Results

### Participation trends and multiple finishes

**Figure 1:**
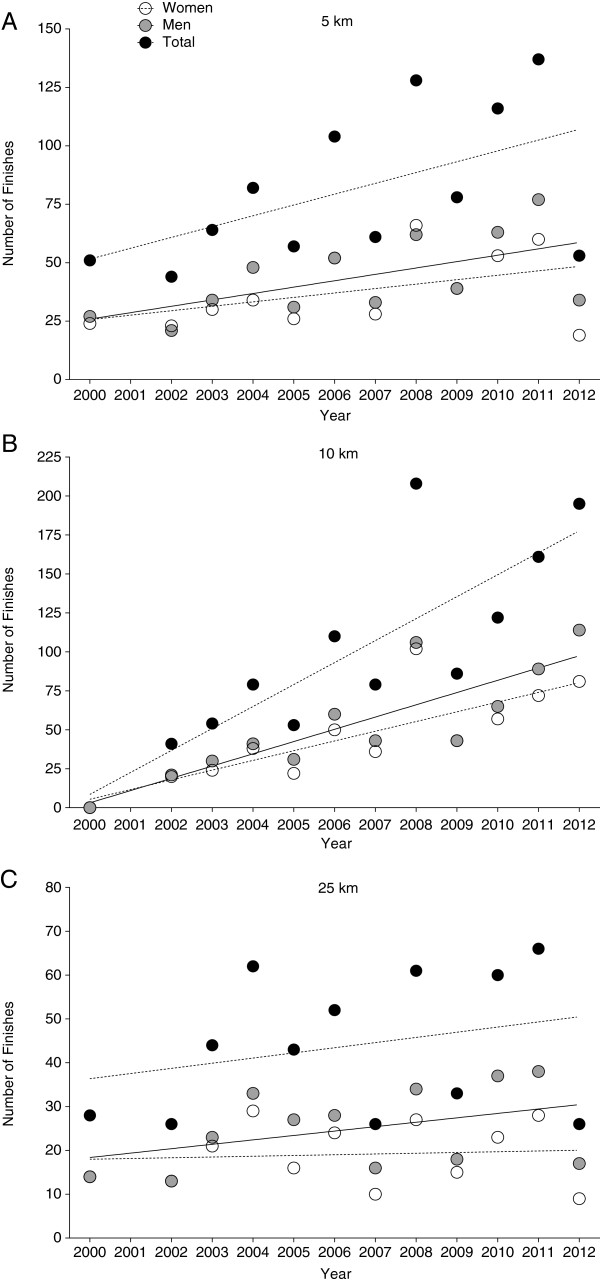
**Annual number of finishers for both sexes. (A)** 5 km **(B)**, 10 km, **(C)** 25 km.

Between 2000 and 2012, the number of finishers in 5 km increased for men (*P* < 0.05), but not for women and overall finishers (*P* > 0.05). In 10 km, the number of finishers increased for both sexes and for overall finishers (*P* < 0.05). In 25 km, the overall number of finishers was constant (*P* > 0.05) (Figure 1). In 5 km, every swimmer finished on average twice, in 10 km approximately 2.6 times, and in 25 km about 2.4 times, respectively (Table 1).

**Table 1:** Number of finishes and multiple finishes for long-distance swimmers in 5, 10 and 25 km

	**Distance**								
	**5 km**			**10 km**			**25 km**		
**Number of finishes**	**Women**	**Men**	**Total**	**Women**	**Men**	**Total**	**Women**	**Men**	**Total**
1 Finish	118	155	273	89	127	215	53	78	130
2 Finishes	54	54	108	36	47	83	10	21	31
3 Finishes	17	22	39	24	27	51	6	8	14
4 Finishes	12	10	22	15	16	31	6	8	13
5 Finishes	5	5	10	13	10	23	5	5	11
6 Finishes	3	6	9	7	5	11	3	6	9
7 Finishes	2	3	5	6	6	13	3	1	4
8 Finishes	2	3	5	3	7	10	1	0	1
9 Finishes	2	1	3	1	1	2	2	1	3
10 Finishes	0	1	1	3	1	4	0	1	1
>10 Finishes	3	2	5	3	6	9	2	3	5
Total									
Finishes	454	521	975	545	643	1,188	229	298	527
Finisher	218	262	480	200	253	452	91	132	222

### Swimming speed and change in swimming speed

**Figure 2:**
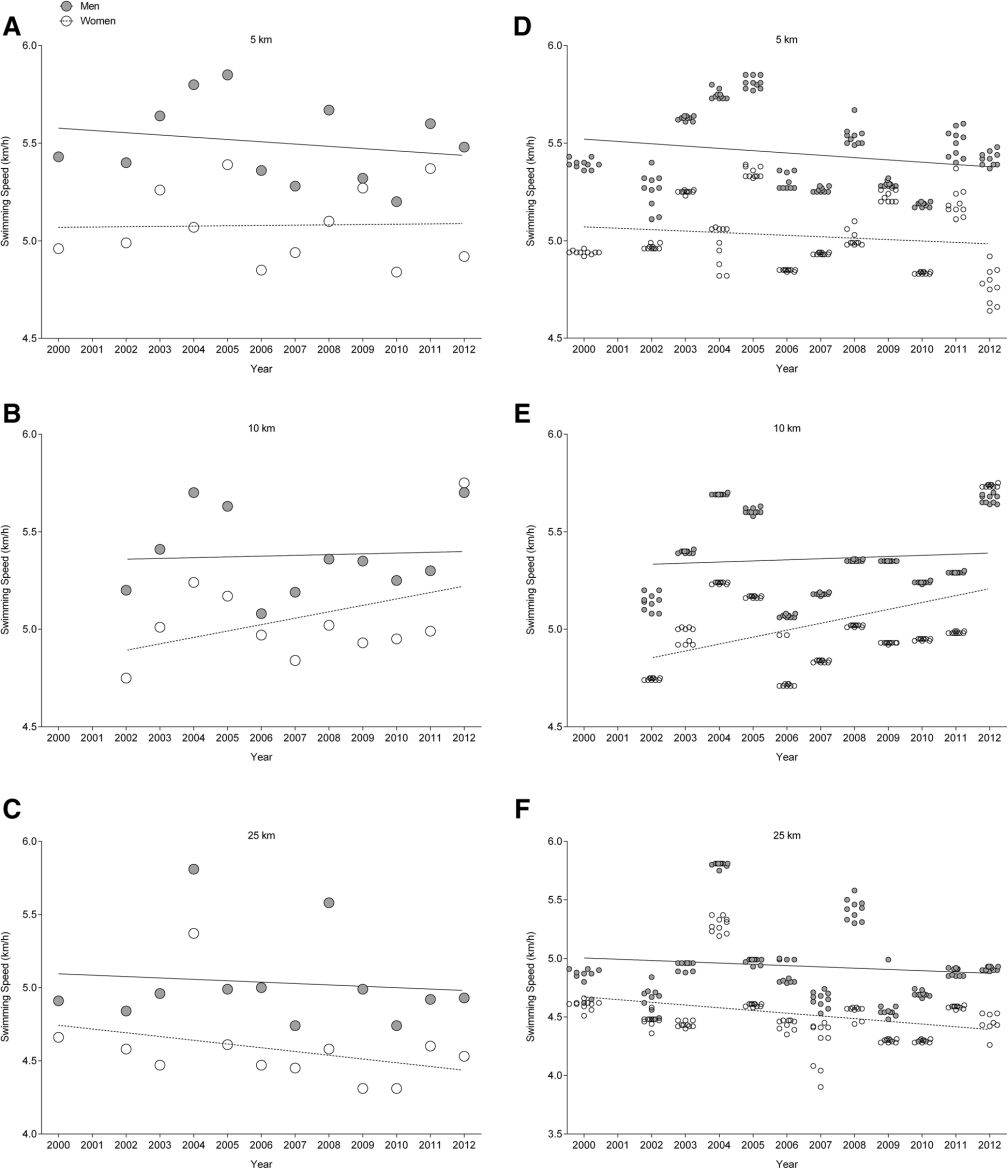
**Average swimming speed.** Average swimming speed of the annual fastest **(A**, **B**, **C)** and the annual ten fastest finishers **(D**, **E**, **F)** for both sexes in 5-, 10- and 25-km swimming events.

The annual fastest men in 10 km increased swimming speed when corrected for multiple finishes and age of athletes with multiple finishes in contrast to women, in whom no changes occurred (Tables 2 and 3, Figure 2). In 5 and 25 km, the annual fastest athletes of both sexes did not improve swimming speed (Tables 2 and 3, Figure 2). For the annual ten fastest swimmers, women increased swimming speed in 10 km and decreased in 25 km, also when corrected for multiple finishes and age of athletes with multiple finishes (Tables 2 and 3, Figure 2). In the annual top ten men, swimming speed decreased in 5 km, also when corrected for multiple finishes and age of athletes with multiple finishes (Tables 2 and 3, Figure 2). In all other events, neither women nor men were able to improve swimming speed.

**Table 2:** Swimming speed and age of the fastest swimmers

	**Distance (km)**	**Annual fastest**		**Annual ten fastest**	
		**Women**	**Men**	**Women**	**Men**
Peak swimming speed (m/s)	5	5.1 ± 0.2	5.5 ± 0.2	5.0 ± 0.2	5.4 ± 0.2
	10	5.1 ± 0.3	5.4 ± 0.2	5.0 ± 0.3	5.4 ± 0.2
	25	4.6 ± 0.3	5.0 ± 0.3	4.5 ± 0.3	4.9 ± 0.3
Age of peak swimming speed (years)	5	22.4 ± 4.6	26.7 ± 3.7	22.5 ± 1.2	24.8 ± 1.0
	10	24.1 ± 4.6	25.6 ± 4.7	23.4 ± 0.9	25.6 ± 1.6
	25	26.8 ± 4.0	27.9 ± 3.8	23.8 ± 0.9	27.2 ± 1.1

**Table 3:** Multi-level regression analyses for swimming speed of the annual fastest swimmers and the annual ten fastest swimmers

	**Model**	***β***	**SE ( *β *)**	**Standard *β***	***T***	***p***
Annual fastest swimmers	5 km, women					
	1	0.002	0.017	0.031	0.097	0.924
	2	0.002	0.017	0.031	0.097	0.924
	3	0.002	0.024	0.037	0.081	0.937
	5 km, men					
	1	-0.012	0.017	-0.210	-0.678	0.513
	2	-0.012	0.017	-0.210	-0.678	0.513
	3	-0.008	0.019	-0.142	-0.413	0.689
	10 km, women					
	1	0.033	0.024	0.409	1.346	0.211
	2	0.033	0.024	0.409	1.346	0.211
	3	0.048	0.038	0.591	1.250	0.247
	10 km, men					
	1	0.004	0.021	0.061	0.183	0.859
	2	0.004	0.021	0.061	0.183	0.859
	3	0.082	0.031	1.278	2.622	0.031
	25 km, women					
	1	-0.026	0.022	-0.352	-1.190	0.262
	2	-0.026	0.022	-0.352	-1.190	0.262
	3	-0.041	0.021	-0.560	-1.926	0.086
	25 km, men					
	1	-0.009	0.027	-0.110	-0.349	0.734
	2	-0.009	0.027	-0.110	-0.349	0.734
	3	0.014	0.032	0.159	0.433	0.676
Annual ten fastest swimmers	5 km, women					
	1	-0.007	0.005	-0.142	-1.559	0.122
	2	-0.007	0.005	-0.142	-1.559	0.122
	3	-0.006	0.005	-0.113	-1.236	0.219
	5 km, men					
	1	-0.012	0.005	-0.214	-2.377	0.019
	2	-0.012	0.005	-0.214	-2.377	0.019
	3	-0.012	0.005	-0.218	-2.424	0.017
	10 km, women					
	1	0.036	0.007	0.421	4.820	< 0.001
	2	0.036	0.007	0.421	4.820	< 0.001
	3	0.035	0.007	0.419	4.796	< 0.001
	10 km, men					
	1	0.006	0.006	0.089	0.929	0.355
	2	0.006	0.006	0.089	0.929	0.355
	3	0.005	0.006	0.073	0.727	0.469
	25 km, women					
	1	-0.023	0.007	-0.312	-3.548	0.001
	2	-0.023	0.007	-0.312	-3.548	0.001
	3	-0.023	0.007	-0.310	-3.498	0.001
	25 km, men					
	1	-0.011	0.009	-0.114	-1.250	0.214
	2	-0.011	0.009	-0.114	-1.250	0.214
	3	-0.011	0.009	-0.120	-1.300	0.196

Model 2 with correction for multiple finishes, and Model 3 with correction for age of athletes with multiple finishes.

### Change in the age of peak swimming speed

**Figure 3:**
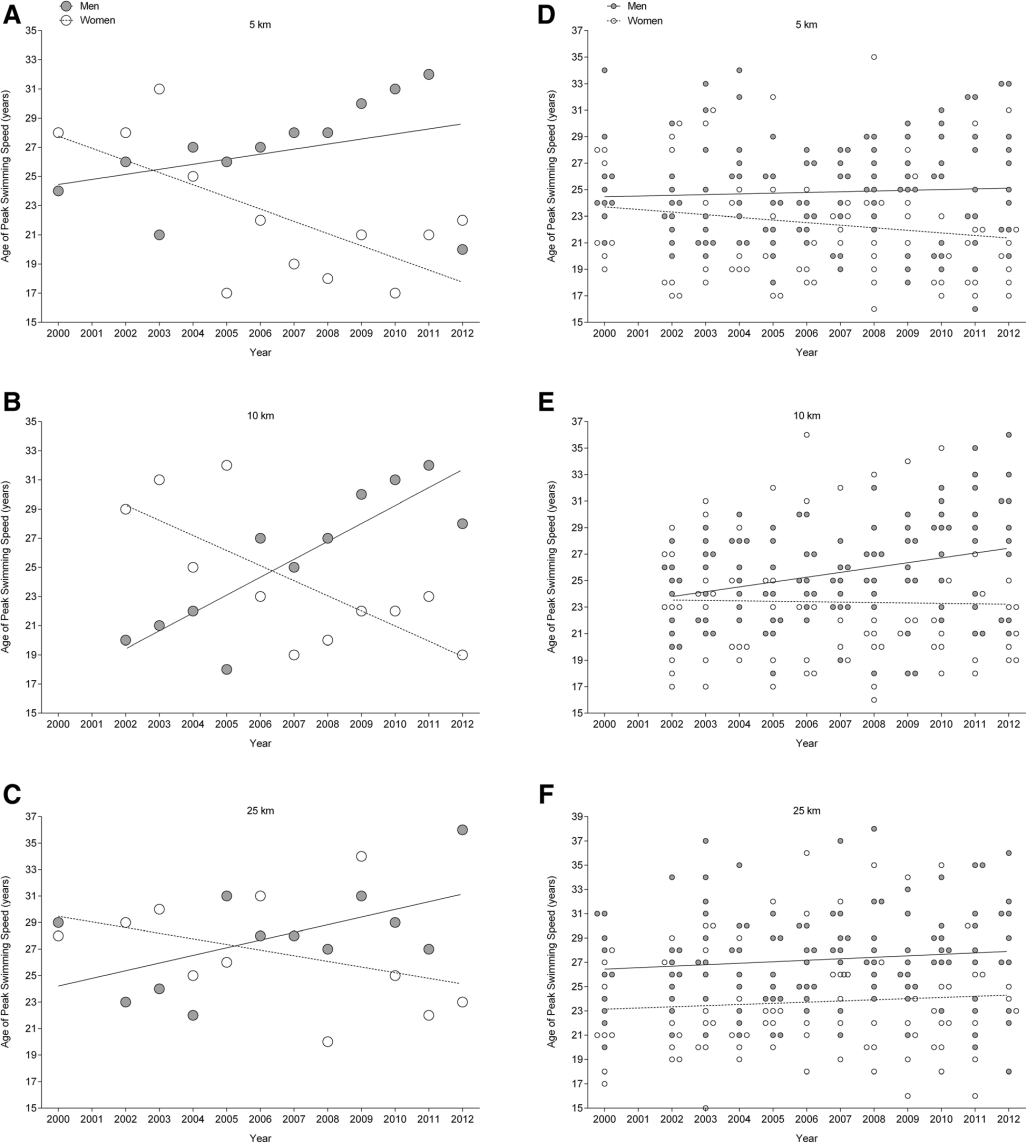
**Age of peak swimming speed.** Age of peak swimming speed of the annual fastest **(A, B, C)** and the annual ten fastest finishers **(D, E, F)** for both women and men in 5, 10 and 25 km.

For the annual fastest swimmers, the age of peak swimming speed decreased for women in 5 km from 28 years to 22 years and in 10 km from 29 years to 19 years, while the age of peak swimming speed was unchanged at 27.0 ± 4.0 years in 25 km, also when corrected for multiple finishes of the athletes (Tables 2 and 4, Figure 3). For men, the age of peak swimming speed was stable in 5 km at 27.0 ± 3.7 years and in 25 km at 28.0 ± 3.8 years, while the age of peak swimming speed increased in 10 km from 20 to 28 years, also when corrected for multiple finishes of the athletes (Tables 2 and 4, Figure 3).

**Table 4:** Multi-level regression analyses for age of the annual fastest and the annual ten fastest swimmers

	**Model**	***β***	**SE ( *β *)**	**Standard *β***	***T***	***p***
Annual fastest swimmers	5 km, women					
	1	-0.833	0.289	-0.674	-2.884	0.016
	2	-0.833	0.289	-0.674	-2.884	0.016
	5 km, men					
	1	0.346	0.288	0.356	1.205	0.256
	2	0.346	0.288	0.356	1.205	0.256
	10 km, women					
	1	-1.036	0.313	-0.741	-3.315	0.009
	2	-1.036	0.313	-0.741	-3.315	0.009
	10 km, men					
	1	1.227	0.240	0.862	5.113	0.001
	2	1.227	0.240	0.862	5.113	0.001
	25 km, women					
	1	-0.424	0.312	-0.396	-1.362	0.203
	2	-0.424	0.312	-0.396	-1.362	0.203
	25 km, men					
	1	0.577	0.268	0.563	2.153	0.057
	2	0.577	0.268	0.563	2.153	0.057
Annual ten fastest swimmers	5 km, women					
	1	-0.194	0.102	-0.172	-1.901	0.060
	2	-0.194	0.102	-0.172	-1.901	0.060
	5 km, men					
	1	0.054	0.098	0.051	0.552	0.582
	2	0.054	0.098	0.051	0.552	0.582
	10 km, women					
	1	-0.032	0.129	-0.024	-0.247	0.805
	2	-0.032	0.129	-0.024	-0.247	0.805
	10 km, men					
	1	0.365	0.109	0.306	3.344	0.001
	2	0.365	0.109	0.306	3.344	0.001
	25 km, women					
	1	0.097	0.106	0.084	0.913	0.363
	2	0.097	0.106	0.084	0.913	0.363
	25 km, men					
	1	0.122	0.103	0.109	1.188	0.237
	2	0.122	0.103	0.109	1.188	0.237

Model 2 with correction for multiple finishes.

**Figure 4:**
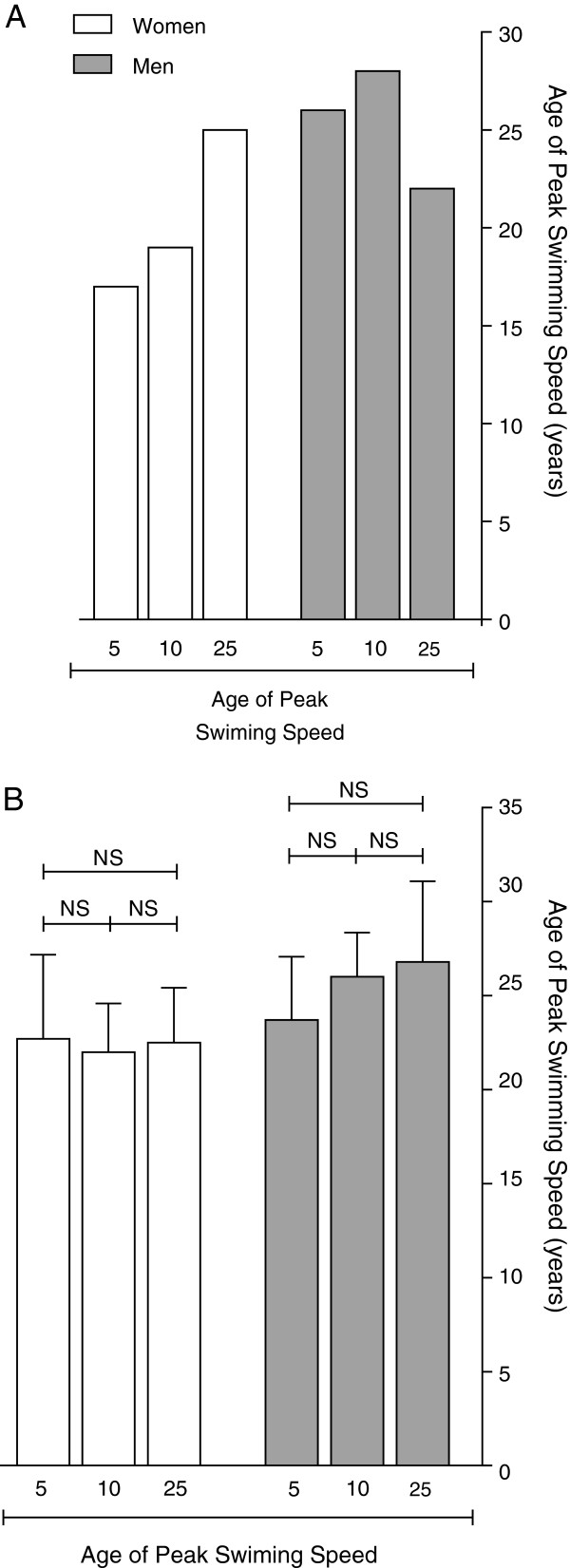
**Age of peak swimming speed.** Fastest swimmer ever **(A)** and of the ten fastest swimmers ever **(B)**.

For the annual ten fastest swimmers in 10 km, the age of peak swimming speed increased in men from 23.7 ± 2.8 to 28 ± 5.1 years, also when corrected for multiple finishes of one athlete, but was stable in 5 km at 24.8 ± 1.0 years and in 25 km at 27.2 ± 1.1 years. In women, the age of peak swimming speed was stable at 22.4 ± 1.2 years in 5 km, at 23.4 ± 0.9 years in 10 km and at 23.8 ± 0.9 years in 25 km (Tables 2 and 4, Figure 3). However, the age of peak swimming speed of the ten fastest swimmers ever was similar between 5, 10 and 25 km at approximately 22 years in women and at about 25 years in men (Figure 4).

### Countries and nationalities

**Figure 5:**
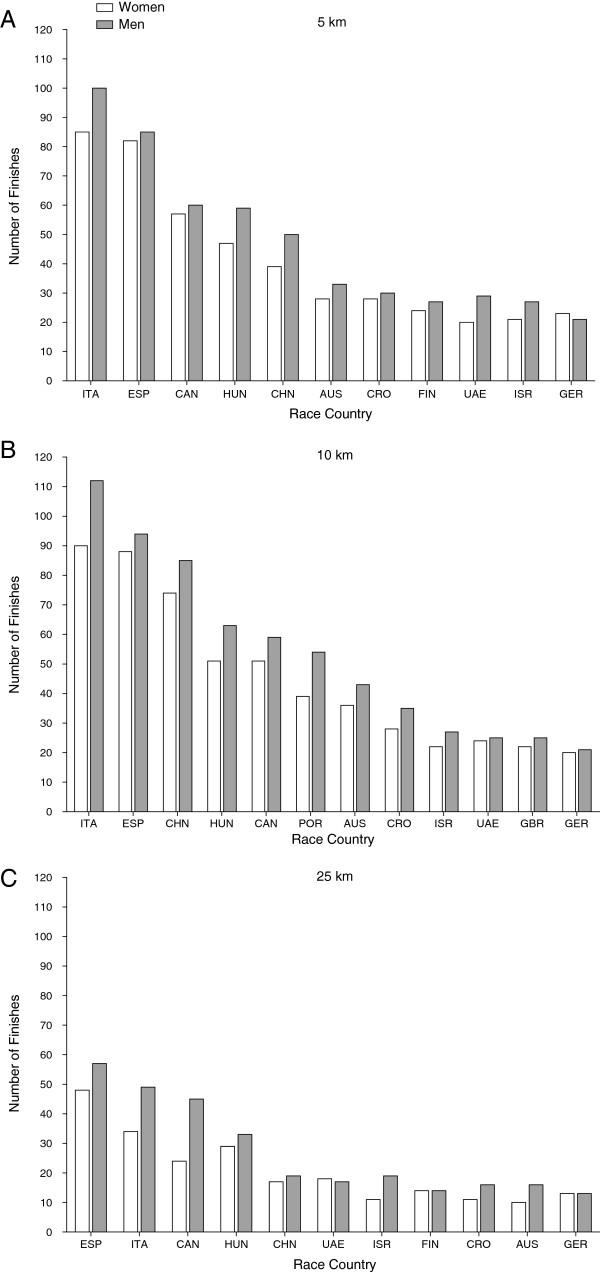
**Number of finishes sorted by the country where the races were held**. ITA = Italy, ESP = Spain, CAN = Canada, HUN = Hungary, CHN = China, AUS = Australia, CRO = Croatia, FIN = Finland, UAE = United Arab Emirates, ISR = Israel, GER = Germany, POR = Portugal, GBR = Great Britain. **(A)** 5 km, **(B)** 10 km and **(C)** 25 km.

**Figure 6:**
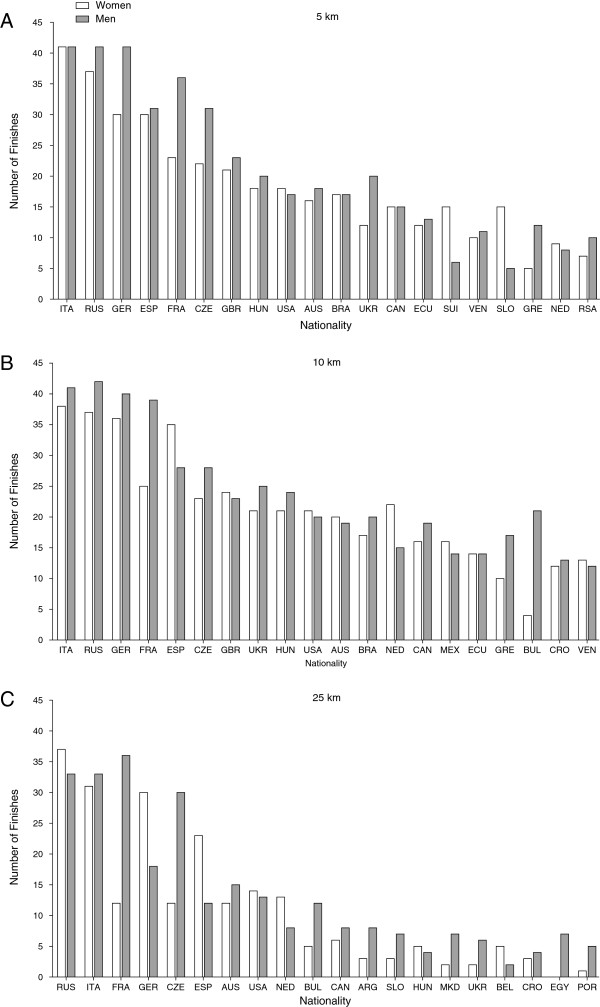
**Number of finishes sorted by the nationality of the swimmers.** Limited to the 20 countries with the highest number of finishes. ITA = Italy, RUS = Russia, ESP = Spain, CAN = Canada, HUN = Hungary, CHN = China, AUS = Australia, CRO = Croatia, FIN = Finland, UAE = United Arab Emirates, ISR = Israel, GER = Germany, POR = Portugal, GBR = Great Britain, BRA = Brazil, UKR = Ukraine, ECU = Ecuador, SUI = Switzerland, VEN = Venezuela, SLO = Slovenia, GRE = Greece, NED = Netherlands, RSA = Republic of South Africa, CZE = Czech Republic, MEX = Mexico, BUL = Bulgaria, ARG = Argentina, MKD = Macedonia, EGY = Egypt. **(A)** 5 km, **(B)** 10 km and **(C)** 25 km.

In 5 and 10 km, most of the finishes were achieved for both women and men in races held in Italy ahead of races held in Spain (Figure 5). In 25 km, however, more finishes were achieved in races held in Spain ahead of races in Italy. In 5 and 10 km, most of the successful finishes were achieved by athletes originating from Italy, Russia and Germany (Figure 6). In 25 km, however, most finishes were achieved by swimmers from Russia, Italy and France.

## Discussion

The main findings were, firstly, that swimming speed plateaued for the annual fastest swimmers in 5, 10 and 25 km for both sexes, except for men in 10 km where it increased. For the top ten swimmers, only women improved swimming speed in 10 km. Secondly, the age of peak swimming speed was approximately 22–28 years over all distances for both the annual fastest swimmer and the annual ten fastest swimmers, and therefore appeared older than those reported for elite freestyle pool swimmers from 50 to 1,500 m [6-10,15].

### Change in swimming speed over the years

Between 2000 and 2012, swimming speed of the annual fastest finishers was unchanged for both sexes over 5, 10 and 25 km, with the exception for men in 10 km where it increased. Therefore, the swimming speed seemed to plateau in 5 and 25 km. The exploration of the limit of human performance has been going on for ages [16-18]. Whipp and Ward [17] suggested that human performance should be unlimited based on linear extrapolations. Other authors reported a plateau effect [18]. In swimming, Nevill et al. [16] analysed the development of swimming world records for men and women over 100-, 200- and 400-m freestyle from 1957–2006. An increase for swimming speeds across all distances was reported during the 1960s and 1970s, while swimming speeds seemed to plateau in the last three decades [16]. The present findings support these results since only men could improve swimming speed in 10 km. However, the present study analysed only 13 years of duration, while other studies analysed longer intervals [9,16]. Therefore, a possible alteration could have been too slight to be detected.

In the ten fastest annual swimmers, only women in 10 km improved swimming speed. Over all other distances, both men and women either decreased or plateaued in their swimming speed. A possible reason for these findings could be the fact that in ultra-distance swimming, the elite was very close together. For example, in the Summer Olympics 2012, Mellouli Oussama from Tunisia won the 10 km in 1 h 49 min 55 s, less than 1 min in front of the next 12 athletes with <1 % time difference. Therefore, solo swims and wins over the whole distance of 5, 10 or 25 km are unheard of, and the main objective in a race may be controlling opponents instead of swimming world records.

A main reason for close results is the fact that open water swimmers are allowed to draft in contrast to pool swimmers [4]. As in cycling, breakaway tries of a single athlete are almost impossible over a larger distance. Influences on swimming speed of water resistance [18,19] in swimming and air resistance in cycling [20,21], and the combination in triathlon [22] have been investigated. Hausswirth et al. [21] reported that continuously drafting behind a leading cyclist allows triathletes to save a significant amount of energy during the bike leg of a sprint triathlon (i.e. 0.75-km swim, 20-km bike, 5-km run). The optimal distance for drafting in swimming was between 0 and 50 cm back from the toes of the lead swimmer, whereas in lateral drafting, the optimal distance was 50–100 cm back from the hands of the leading swimmer [23,24]. The resistance in a medium increases in a quadratic manner when the moving speed doubles [25]. Since the density of water is far above the density of air, drafting plays an important role even at slower swimming speeds. Therefore, drafting [26] may be the main difference of long- to short- and middle-distance swimming.

### The age of peak swimming speed in long-distance swimmers

The age of peak swimming speed increased with increasing race distance from 5 to 25 km in both sexes. However, the age of peak swimming speed did not differ significantly among 5, 10 and 25 km. The fastest women were approximately 24 years old, and the fastest men were about 26 years. While the age of peak swimming speed decreased from 50 to 1,500 m freestyle in pool swimmers [6,9], it increased in longer race distances such as 26.4 km [11]. These results were in line with previous findings of Eichenberger et al. [11-13], where the age of peak swimming speed was 30–39 years for 26.4 km, 12 h and 35 km compared to approximately 21 years described by Berthelot et al. [6] in 50 to 1,500 m freestyle swimming. However, Berthelot et al. [6] reported differences in the age of peak swimming speed for swimmers regarding the length of a swimming performance. The 1,500-m freestyle was achieved at a younger age of about 18 years compared to the 50-m freestyle at approximately 23 years.

Combined with the results of the present study, it seems likely that the age of peak swimming speed decreased from about 23 years in short-distance sprint swimming of 50 m to approximately 18 years in 1,500-m long-distance swimming to increase again from to about 27 years in 25 km. Recent investigations [27,28] addressed the possible association of the age of peak performance and the length of a race, i.e. the distance of an event. In running, for example, Rüst et al. [28] mentioned the possibility that older runners rather compete in ultra-marathons because of a deficit in physiological factors as maximal oxygen uptake (VO_2_max) [29] compared to young athletes in their ‘prime age’. It could be argued that the world's elite swimmers rather compete in the more lucrative short- and middle-distance than in long-distance events.

Physiological and anthropometric differences might explain the differences in the age of peak swimming speed for short, middle and long distances. Peak swimming speed in sprint swimming was highly associated with strength, power [30] and anaerobic capacity [31]. In longer race distances such as 1,500-m freestyle swimming, peak swimming speed was rather associated with maximal oxygen consumption [31], anaerobic threshold [32] and anthropometric characteristics such as body fat [33-35]. In contrast to other sports disciplines such as running [9] or triathlon [27], the age of peak performance seemed to decrease in swimming [9]. The finding that the age of peak swimming speed increased again from 5 to 25 km therefore is a new finding. This finding could have an impact on aging athletes as they could focus on longer swimming distances in the future as elite athletes do in other sports such as running [28].

Another important aspect is motivation to compete in different swimming events. While short- (50 m) to middle-distance (1,500 m) swimming stars such as Michael Phelps or Ryan Lochte earned hundreds of thousands to millions of dollars [36], the average elite swimmer lived on much less. Indoor Olympics swimming disciplines attract huge publicity as even TV transmission times were adjusted to the main marketing markets in the 2012 Olympic Games in London. In contrast, long-distance swimming such as 5, 10 and 25 km is little known to the public, and 5- and 25-km events are not held at the Olympic Games [5]. Short- and middle-distance swimming were two of the major attractions in the last Olympic Games in Athens (2004), Beijing (2008) and London (2012). Swimming was represented in 34 different competitions (i.e. each sex 17) providing for 102 medals of a total of 992 Olympic medals 2012 [5], only the two 10-km marathon swims were longer than the 1,500-m indoor freestyle. Therefore, it could be argued that long-distance swimming was less attractive to the world's elite swimming athletes. Furthermore, less publicity might have an influence on professionalism of training in young swimmers, which could have an important influence in the age of peak swimming speed.

Most of the races were held in European countries, and mainly European swimmers were competing in these races. Although American and Australian athletes dominate pool swimming [37,38], European athletes seemed to dominate in ultra-distance races such as swimming [11,39], cycling [40], running [41-44] and triathlon [45-48].

### Limitations and implications for future research

The strength of the study is that the statistical analysis excluded the influence of athletes who competed more than once and could have biased the results. Furthermore, this study is the first to investigate the association of age and peak swimming performance in long-distance open water swimming so far about professional swimmers competing in FINA races. The study is limited since variables such as physiological parameters [23], anthropometric characteristics [35], training data [49], previous experience [50], nutrition [51,52] and motivational [53] factors were not considered. These variables may have had an influence on race outcome. We used a linear regression analysis; however, the sex difference is considered to follow a non-linear change [54]. Further studies need to investigate why the upper limit of swimming speed but not the increase of density is reached in long-distance open water swimming. Moreover, a systemic analysis of age of peak swimming speed across distances from 50 to 25 km and more would be useful. Future studies need also to investigate why European athletes preferably compete in ultra-distance swimming races.

## Conclusion

The upper limit of peak swimming speed of the fastest annual swimmers plateaued in long-distance open water swimming up to 25 km as only women in 10 km increased swimming speed. The main focus of elite long-distance swimmers seemed to lie more in controlling opponents than accomplishing new records. The age of peak swimming speed seemed older (approximately 25 years) for long-distance swimmers compared to the age of freestyle swimmers in 50- to 1,500-m races (about 20–23 years). Future studies need to investigate why short- and middle-distance swimming regularly produced world best times, while long-distance swimming speed seemed to plateau. Furthermore, the differences in physiology and anthropometry such as body fat for short-, middle-, and long-distance swimmers need more investigation.

## Abbreviations

ARG: Argentina; AUS: Australia; BRA: Brazil; BUL: Bulgaria; CAN: Canada; CHN: China; CRO: Croatia; CZE: Czech Republic; ECU: Ecuador; EGY: Egypt; ESP: Spain; FINA: Fédération Internationale de Natation; FIN: Finland; GBR: Great Britain; GER: Germany; GRE: Greece; HUN: Hungary; ITA: Italy; ISR: Israel; MEX: Mexico; MKD: Macedonia; NED: Netherlands; POR: Portugal; RSA: Republic of South Africa; RUS: Russia; SLO: Slovenia; SUI: Switzerland; UAE: United Arab Emirates; UKR: Ukraine; VEN: Venezuela.

## Competing interests

The authors declare that they have no competing interests.

## Authors' contributions

MAZ drafted the manuscript. BK collected the data and helped draft the manuscript. CAR added statistical analyses and helped draft the manuscript. TR participated in the study design and helped draft the manuscript. RL helped draft the manuscript. All authors read and approved the final manuscript.
